# Characterizing symptoms of e-cigarette dependence: a qualitative study of young adults

**DOI:** 10.1186/s12889-021-10945-z

**Published:** 2021-05-20

**Authors:** Kelsey A. Simpson, Afton Kechter, Sara J. Schiff, Jessica L. Braymiller, Naosuke Yamaguchi, Rachel Carmen Ceasar, Ricky N. Bluthenthal, Jessica L. Barrington-Trimis

**Affiliations:** 1 Department of Preventive Medicine, Keck School of Medicine, University of Southern California, 2001 N. Soto Street, Los Angeles, CA 90089 USA; 2 Institute for Addiction Science, University of Southern California, Los Angeles, CA USA

**Keywords:** E-cigarette, Dependence, Young adults, Nicotine, Addiction, Qualitative

## Abstract

**Background:**

While rates of e-cigarette use (‘vaping’) continue to potentiate concern, there is limited data on common symptoms of e-cigarette dependence among young adults who vape. This study sought to critically explore how young adults experience, manifest, and conceptualize vaping dependence symptoms in their everyday lives.

**Methods:**

Between June 2018 and 2019, in-depth qualitative interviews were conducted with 62 young adults who use e-cigarettes (aged 18–25) and live in Southern California. We explored participants’ product preferences, daily e-cigarette use patterns, vaping history, withdrawal experiences, and quit attempts or periods of cessation. We used a thematic analysis approach to interpret the transcripts.

**Results:**

Young adults discussed nine dimensions of vaping dependence that were organized into two categories: 1) general nicotine dependence symptoms, and 2) unique dependence symptoms related to vaping. Nicotine dependence symptoms included cravings and urgency to use, increased use to achieve desired effects, and unsuccessful quit attempts and withdrawal. Symptoms unique to vaping dependence included greater nicotine consumption due to accessibility and lack of restrictions, habitual vaping, inability to track vaping frequency, immediate gratification and comfort, social acceptability and norms, and awareness of vaping dependency.

**Conclusions:**

In addition to nicotine dependence symptoms that have been characterized for other tobacco products, young adult e-cigarette users described unique symptoms of vaping dependence that necessitate the need for more refined measures. All dimensions of vaping dependence should be considered in discussions of policies as well as treatment and education efforts intended to protect young people from e-cigarette dependence.

**Supplementary Information:**

The online version contains supplementary material available at 10.1186/s12889-021-10945-z.

## Introduction

Despite nationwide prevention efforts, the prevalence of e-cigarette use (i.e., ‘vaping’) among youth and young adults remains high, with more than 1 in 5 youth and more than 1 in 6 young adults (aged 19–28 years) reporting past 30 day use in 2019 [1, 2]. The high levels of nicotine in e-cigarettes that are popular among young people – for example, those using highly concentrated nicotine salt solutions – are a cause for concern [3–5]. Nicotine salt solutions have been shown to increase rates of nicotine absorption and produce vapor that is less harsh and more palatable than free-base nicotine, which allows for highly concentrated nicotine solutions that may facilitate repeated use and addiction [6–8]. The potential for e-cigarettes to evoke symptoms of nicotine dependence has not been extensively studied, particularly among young adults. Given the popularity of e-cigarettes among this age group and increased levels of nicotine in such products, research evaluating the unique risk of nicotine dependence associated with vaping is needed.

To date, the majority of research on nicotine dependence has occurred in the context of conventional cigarette use. Research on e-cigarette dependence is more nascent [9–13]. For example, a quantitative study comparing adolescent pod users (i.e., those using e-cigarettes that contain pre-filled nicotine-containing pods) versus non-pod users (i.e., those using other types of e-cigarettes without pre-filled pods) found that pod users exhibited higher levels of self-reported nicotine dependence than non-pod users [12]. Further, in a mixed-methods study examining perceptions of JUUL and nicotine dependence, many adolescent JUUL users expressed feeling ‘addicted’ to their JUUL but not nicotine, and often lacked awareness of both the dangers of using nicotine and of the high levels of nicotine in their e-liquid solutions [11]. Additionally, a text analysis of tweets related to nicotine effects, dependence, and withdrawal among JUUL users found that those who vaped discussed strong physiological withdrawal symptoms due to e-cigarettes, desires to quit vaping, and accounts of feeling ‘addicted to’ or ‘dependent on’ their JUUL device [13]. While these findings provide initial evidence that some individuals who use e-cigarettes may experience dependence, research among young adults is sparse. Moreover, the studies available have generally utilized surveys that were originally validated in samples of primarily adult, combustible cigarette users, and which do not address the potentially unique expression of nicotine dependence that may occur for those who vape [14, 15]. This has thwarted researchers’ ability to discern the product-specific characteristics of e-cigarettes that may potentiate the risk for dependence.

Accordingly, the main objective of the present work was to expand upon the current literature by examining young adults’ experiences and accounts of e-cigarette dependence directly, using qualitative interviews to elicit information beyond symptoms of dependence commonly reported for traditional tobacco users. We aimed to better understand how young adults who vape experience, manifest, develop, and conceptualize symptoms of dependence in their everyday lives. Findings are critical to understanding both the public health impact and disease burden that may result from rapid increases in e-cigarette use, as well as informing the design of new measures to identify and prevent vaping dependence that are better tailored to young adults’ specific needs and experiences.

## Methods

### Study population and recruitment

Data were from a qualitative study of young adults who use e-cigarettes (ages 18–25, *N* = 62). Participants were recruited using posted fliers and online advertisements on public and social media platforms (i.e., Twitter, Instagram, Facebook, Craigslist), and were interviewed between 06/2018 and 06/2019 at our research facilities in Los Angeles. Initial respondents were screened for eligibility through online surveys and over the phone with study staff. To be included in the study, participants had to be 18 to 25 years old and self-reported to have vaped on a weekly basis or more for at least 5 months prior to study enrollment. Written informed consent was obtained from each participant prior to enrollment, and all subjects were compensated $50 USD for their participation. All study procedures were approved by the University of Southern California (USC) Institutional Review Board.

### Procedure

One-on-one qualitative interviews were conducted by study staff (KS, AK, and SS). Interviews were conducted face-to-face using a semi-structured interview guide in a private designated study room at USC. Qualitative interview guide topics included: e-cigarette use patterns, preferences for different tobacco and alternative tobacco products, withdrawal symptoms, quit attempts or periods of cessation, as well as other topics such as family substance use, education, jobs, and more (complete interview guide available upon request). Interviews ranged in duration, lasting approximately 30 to 90 min. Upon completion of the qualitative interview, participants completed a short quantitative survey electronically.

### Quantitative measures

Items on the quantitative survey included age (continuous), race/ethnicity (American Indian or Alaska Native, Asian, Black or African American, Native Hawaiian or Pacific Islander, White, Multiethnic/Multiracial, Hispanic or Latino, Other), gender (male/female), past 30-day vaping or combustible cigarette use frequency (separately; 0, 1–5, 6–19, or 20 or more days), dual e-cigarette/combustible cigarette use (yes/no), any vaping quit attempts (yes/no), current subjective financial status (don’t meet basic expenses, just meet basic expenses, meets needs with a little left, live comfortably), and current enrollment in higher education (yes/no/don’t know). The Adapted Hooked on Nicotine Checklist was also used to measure electronic nicotine dependence [16] (indicated by a positive screen of 1 or more dependence symptoms). Primary participant device type (JUUL, other pod-product, mods) was based on photos of each participant’s device, collected during the interview.

### Statistical analysis

Sessions were audio-recorded and transcribed verbatim. Transcripts were then imported onto NVivo (Version 12.5) where they were systematically organized and analyzed using an iterative, multi-step process. First, the entire investigative team independently read through a set of transcripts and wrote summary memos, which were discussed with the study team. An initial set of codes (“vaping transitions,” “access to vaping products,” “vaping dependence,” and “vaping terminology”) were developed to describe core concepts that emerged from the transcripts, which were independently coded by two team members in sets of 5 until adequate consensus was reached (Kappa > 0.70). The current paper focused on the “vaping dependence” code. The team developed an additional set of emergent themes by reviewing quotations initially coded under vaping dependence, ranking the most important quotes, and discussing core concepts, patterns, and relationships across transcripts. This process occurred over the course of several team meetings, and a final set of themes related to vaping dependence were established.

## Results

### Participant characteristics

The analytic sample included 62 participants, which have been described previously [17]; see Table 1. Briefly, the sample was majority male, racially and ethnically diverse, about 21 years of age at the time of the survey, and living within comfortable financial means. The sample was split between those who only used e-cigarettes, and those who used both e-cigarettes and combustible cigarettes. Three quarters of our sample reported trying to quit or cut down on vaping at some point (75%), and the majority of these respondents reported nicotine dependence symptoms (87%). Overall, the sample used a variety of device types including JUUL (33.9%), other pod-based products (22.6%), mods (25.8%); 11 participants (17.7%) did not bring their device to the interview.

**Table 1:** Sociodemographic and substance use characteristics of total sample of young adults who vape, aged 18–25 (*N* = 62)^**a**^

Characteristic	N (%) or M (SD)
**Gender, N (%)**	
Male	49 (79.0%)
Female	13 (21.0%)
**Race, N (%)**	
American Indian or Alaska Native	2 (3.4%)
Asian	10 (16.9%)
Black or African American	5 (8.5%)
Native Hawaiian or Pacific Islander	2 (3.4%)
White	35 (59.3%)
Multiethnic or Multiracial	7 (11.9%)
Other	8 (13.6%)
**Ethnicity, N (%)**	
Hispanic/Latino	16 (26.7%)
Non-Hispanic/Latino	44 (73.3%)
**Age, M (SD)**	20.9 (1.3)
**Currently enrolled in higher education, N (%)**	
Yes	33 (55.0%)
No	24 (40.0%)
Don’t know	3 (5.0%)
**Current subjective financial status, N (%)**	
Don’t meet basic expenses	1 (1.7%)
Just meet basic expenses	13 (21.7%)
Meets needs with a little left	24 (40.0%)
Live comfortably	22 (36.7%)
**E-cigarette/combustible cigarette user, N (%)**	
Sole e-cigarette user	31 (50.0%)
Dual e-cigarette/combustible cigarette user	30 (48.4%)
**Past 30-day combustible cigarette use, N (%)**	
0 days	12 (28.6%)
1–5 days	18 (42.9%)
6–19 days	8 (19.0%)
20 or more days	4 (9.5%)
**Past 30-day number of days vaped nicotine product, N (%)**	
0–5 days	7 (11.7%)
6–19 days	12 (20.0%)
20–29 days	13 (21.7%)
All 30 days	28 (46.7%)
**Ever tried to stop or cut down use of electronic nicotine devices, N (%)**	
No	15 (25.0%)
Yes	45 (75.0%)
No nicotine dependence symptoms^b^	6 (13.3%)
Any nicotine dependence symptoms^b^	39 (86.7%)
**Primary Device Used**^**c**^	
JUUL	21 (33.9%)
Other pod-based product	14 (22.6%)
Mod	16 (25.8%)
Missing^d^	11 (17.7%)

^a^Available data Ns for denominator ranged from 45 to 62; ^b^Based on the Hooked on Nicotine Checklist, administered only among those who answered “yes” to having ever tried to stop or cut down use of e-cigarettes; ^c^Based on photo of device the participant brought to interviews; ^d^No device was brought to interview

### Thematic analysis

Participants discussed nine dimensions of e-cigarette dependence which we organized into two main categories: themes that align with nicotine/tobacco dependence more broadly (Cravings and urgency to use; Increased quantity and frequency of use to achieve desired effects; Unsuccessful quit attempts and withdrawal symptoms), and themes unique to e-cigarette dependence (Greater nicotine consumption due to ease of accessibility and lack of vaping restrictions; Habitual vaping; Inability to track vaping frequency; Immediate gratification and comfort from vaping devices; Social acceptability and changing norms; Awareness of vaping dependency). See Supplemental Table 1 for additional supporting quotations.

### Themes that align with nicotine and tobacco dependence

#### Cravings and urgency to use

Young adults who vape reported experiencing strong cravings and urges to vape in situations when they were unable to do so. Such cravings were linked to continued e-cigarette use in order to feel relieved or satiated. Several narratives illustrate the contexts in which these experiences occurred:

*It’s crazy because we call “fiending” when you just really want to hit something and you can’t. I’ve really felt that a lot where I get irritable like damn I really wish I had my vape right now…and there’s this overwhelming satisfaction when you do hit it after a long day or you haven’t hit it in a while - it’s dangerous how good it feels. [ID02, Asian, male, age 19]*

*There was a period where I really craved the Suorin [vaping device] because it became a thing where if I was stressed during classes or during an essay or something or some homework, I would definitely take a puff, get lightheaded, feel my whole body relax… [ID25, Hispanic/Latino, male, age 18]*

#### Increased quantity and frequency of use to achieve desired effects

Participants described their propensity to escalate the quantity and frequency of their usage in order to feel the same ‘head buzz’ or sense of ‘normalcy.’ For example:

*Once you start getting used to these pods, you’re going to need more to get to that same level. So, you’re just constantly running through them faster and faster. [ID35, white, male, age 20]*

*I think by the end of that semester it became less of something I did to “release stress” because of the head buzzes and it was just something I needed to do for a sense of normalcy. [ID11, Asian, male, age 20]*

Other participants reflected on the progression in the dosage of nicotine concentration in their e-liquids since starting vaping:

*I have 50 [mg] cause I started with the 30 and then I was like let me go up and see if I can feel any more lightheadedness and I do. [ID37, African American, male, age 25]*

*Before I was just doing it just for fun. But now I was like oh that’s kind of cool, so I started at maybe 3 mg per mL and then eventually [increased to] 6 mg per mL. [ID53, Hispanic/Latino female, age 22]*

#### Unsuccessful quit attempts and withdrawal symptoms

Many participants described their experiences trying to quit using e-cigarettes and failing to do so due to overriding emotional and physical withdrawal symptoms. One participant described his quest to quit vaping on a family vacation and the disappointment that followed:

*This past summer… I tried quitting on a family vacation…I was really killing the vibe for the rest of my family so I went two full days without [vaping] and honestly on that second day I was like I know that I can do this. I know that I can drop it, but I don’t know how to stop myself from being irritated and angry when I’m off of it and I know that I’m really upsetting the rest of my family…I accepted that the day I quit will have to come another day and then I just [vaped] so I could enjoy the vacation. [ID11, Asian, male, age 20]*

Other participants expressed intentions to quit vaping, but also had negative expectations about the effects of nicotine withdrawal on the body:

*I rip it so many times throughout the day that I am scared to stop ...my blood composition has got to have nicotine in it. Like a lot of nicotine. I’m going to go through some sort of withdrawals if I try to cold-turkey it or wean myself off of it less and less. [ID61, white, female, age 19]*

Participants in our sample described specific vaping-induced withdrawal symptoms and the impacts of those symptoms on daily life functioning:

*I get headaches, I get more irritated, it gets a little harder for me to concentrate in class. [ID11, Asian, male, age 20]*

*Sometimes if I have difficulty sleeping and I know I’ve been using it more, I’ll be like “it’s probably the nicotine withdrawal” and I’ll hit it once and go to bed. [ID32, Asian, female, age 21]*

### Themes unique to e-cigarette dependence

#### Greater nicotine consumption due to ease of accessibility and lack of vaping restrictions

Young adults who reported previously smoking combustible cigarettes and transitioning to e-cigarettes described consuming more nicotine with e-cigarettes as opposed to conventional cigarettes. Participants often attributed this to the lack of vaping restrictions relative to cigarette smoking restrictions in public:

*I definitely consume more nicotine than I would if I was smoking cigarettes because I can vape inside, can vape everywhere. [ID13, white, male, age 22]*

*When I started [the Juul] I just found myself using it all the time. With the cigarettes, at least I’d have to get myself up, go outside, light the cigarette, and then put it out and then walk back in, it’s a whole process. I could just be sitting there doing homework and I’m just smoking [vaping], and I’m like hold up, this is supposed to help me quit. [ID01, male, age 20]*

Some described sleeping within close proximity to their vape device in order to use it immediately upon waking up:

*The worst thing is like - first thing in the morning, it’s under my pillow. This is my [rock] bottom, it’s under my pillow [when I sleep so I can use it] first thing in the morning. [ID61, white, female, age 19]*

*I wake up and I hit my vape. I sleep next to it. [ID34, white, male, age 23]*

#### Habitual vaping

Participants described their e-cigarette use as automatic, where their use was oftentimes so ingrained in their daily life functions that they were often unconscious of actually using their e-cigarette. For example:

*You sit there and you mindlessly puff. You get such a high nicotine intake that you’re growing accustomed to [it]. Even if you’re a pack a day smoker, you’ll smoke a whole pod and a half in a day or something. It’s a lot. [ID01, male, age 20]*

*You use it [vape] to a point to where it’s like muscle memory, so like you’re doing homework or relaxing and I’d find myself wanting to reach for it. [ID30, white, male, age 20]*

Participants commonly described their daily use patterns as constant, and the majority of users reported having their e-cigarette on them throughout all hours of the day. For example:

*I kind of just carry it around…before class, after class, in between class, like right after meals especially and right before I sleep. It’s just out of habit…I’m hitting it constantly. [ID11, Asian, male, age 20]*

*I try not to start until later in the day like I’ll try to hold off until I like can’t. It’s usually like 11-12ish around mid-day and then I usually Juul on my way to class, between classes, every time I exit a building and I’m walking in open air. [ID01, male, age 20]*

#### Inability to track vaping frequency

When evaluating an individual’s level of dependence on a substance, assessing the quantity and frequency of use is an important indicator to consider [18]. In the context of e-cigarette use, this is an increasingly challenging thing to measure due to the variability in device types, nicotine concentrations, and puffing/vaping behaviors. Participants in our sample described the dangers attributed to unknown standards for measuring the nicotine content and amount they’re vaping. For example:

*I wish that I was able to measure how much I was [vaping]. I have no idea how much nicotine I’m taking in. I really don’t. I always have it, so I don’t even really know how often I’m ripping it. [ID61, white, female, age 19]*

#### Immediate gratification and comfort from vaping devices

Participants compared the immediate gratification and comfort they feel from vaping to the common relationship they perceive their peers to have with cell phones in the sense that they always have it on them and can pull it out of their pockets to use in order to fill a void – referring to both as adult pacifiers.

*When I’m in class, when I need to be distracted, when I’m feeling anxious and like literally, anything, if I pick up my Suorin [vaping device] it’s like an adult pacifier. I feel like in our society, I have a bad habit, I chew a lot of gum, I rip that nicotine device, and checking your phone. And that somewhere between those three things in my life, it’s like one of those is constantly going on. Just an immediate gratification. [ID61, white, female, age 19]*

Another participant discussed the sense of comfort and safety he felt from vaping:

*I just think there’s something about the oral fixation, like I always kind of jokingly call it like the adult pacifier. Like that’s what the Juul is. It’s like, it’s just something we put to our mouths because it makes us feel safe. [ID58, white, male, age 22]*

#### Social acceptability and norms impact e-cigarette use patterns

Many described the influence of social norms and the acceptability of vaping as a factor that influences continued use. For example, desirable social aspects of vaping were described in the process of learning vape tricks and sharing on social media and with peers who also engage in such practices:

*I think I’m more addicted to nicotine than I am to vaping because I feel like vaping is the social aspect too. It’s kind of like taking pride in that you’re showing off this device that you have, kind of like a new phone. [ID51, Hispanic/Latino, male, age 20]*

Another participant described a vaping game played at parties that illustrates how social influences can facilitate increased exposure to nicotine and heavier patterns of use:

*There’s a game called baseball where a whole party is like a tap Juul and you pass it to one person. They inhale, keep it inside – Pass it to me. I inhale, pass it back, they exhale. Hit again and we just keep going and the longer you have it inside, the more you absorb so then someone quits and is like “I’m done” and the other person wins or whatever. So, yeah, it’s really intense. [ID25, Hispanic/Latino, male, age 18]*

Another participant described her difficulty in quitting vaping due to social pressures:

*[The hard part is] trying to quit [among] a group of friends [who vape]. We all can [quit if], we can all be isolated and be by ourselves for three weeks... It wouldn’t be hard to quit and just stop using all together, but then the social aspect of it comes into play. [ID32, Asian, female, age 21]*

#### Awareness of vaping dependency

Many demonstrated a clear recognition of their vaping dependency, and the implications of that on their identity:

*[Vaping] is something that I’ve integrated into like everything that I do. There are posts on the internet that say how do you know that you’re addicted to vaping, and it’s like [vaping] becomes a part of you, like you look down and you realize it’s in your hand, you don’t even know. [ID20, white, male, age 21]*

*I kind of gave in to that addiction and got onto it [vaping] full-time, and I’ve not gone a day without it since last April, which is pretty weak. It’s in my hand all the time, even in photos and stuff. [ID61, white, female, age 19]*

## Discussion

The results of this study provide novel insights into the characterization of e-cigarette dependence among young adults who vape. Some of the emergent themes discussed by participants resembled indicators of nicotine dependence similar to those studied in the context of combustible cigarettes. Others represented unique facets of e-cigarette-specific dependence (arising from device characteristics and/or the environment created by e-cigarettes). Findings hold important implications for the future development of assessments of vaping dependence, and interventions to reduce vaping dependence among young adults.

Young adults repeatedly described nicotine dependence symptoms that are often reported by cigarette smokers. Many described strong cravings and urges to vape that were alleviated by continued vaping, a similar paradigm to that observed for cigarette smokers who similarly report strong cravings and urges which are alleviated by smoking. Given the literature acknowledging craving and urges to use nicotine as a critical precipitant of smoking relapse outcomes [19] and current diagnostic importance of craving in the characterization of tobacco use disorder in the DSM-V, [20, 21] future studies on vaping dependency should include e-cigarette craving measures such as the Questionnaire of Vaping Craving (QVC) [22] as an important indicator of risk.

Participants frequently discussed unsuccessful attempts to quit vaping and uncomfortable withdrawal symptoms during periods of abstinence or sustained use, which have been reported previously [13, 23–25]. Despite the report of similar withdrawal symptoms to those using other tobacco products [26–28], the degree and severity in which young adults who vape experience withdrawal symptoms due to e-cigarettes is not well established [18]. Given that pharmacological withdrawal from nicotine is a strong marker of dependence in tobacco smokers [8], future studies should continue to track and monitor withdrawal symptomatology in e-cigarette users.

Young adults in our sample reported using their e-cigarette first thing in the morning, which is again consistent with previous research on nicotine dependence both among e-cigarettes users [13], and among other nicotine product consumers [15, 29]. While time to first cigarette is a well-validated predictor of physical dependence to nicotine in tobacco smokers [30], youth may not report using a product within 60 min of waking, possibly due to inability to use a product before leaving the house (away from parents or guardians). However, given newer designs of e-cigarette products that produce far less vapor than earlier generation products, more youth may act on their inclination to use products soon after waking, indoors, without detection. Strikingly, a number of individuals indicated sleeping within close proximity of their vape, and some reported keeping their vape under their pillow at night. In combination with reports from participants regarding early morning use, there is potential utility in continuing to assess time to first vape, which may be indicative of early vaping dependence and abuse potential. In addition, another historically common symptom of dependence is escalation in use; this study revealed that young adults who vape often reported escalations in the frequency and quantity of their e-cigarette use over time. Participants also discussed developing a tolerance to nicotine, which was reflected by increases in the amount needed to vape in order to feel the same physiological effects of nicotine they felt previously.

Other aspects of dependence emerged from this study that have not been previously described in research on nicotine dependence through traditional tobacco products. Participants described social aspects of vaping that included pride associated with showing off one’s vaping device, difficulty quitting due to social pressures, and enjoyment derived from games played at parties where the winner is chosen based on vaping tolerance. These rituals and norms for using e-cigarettes likely contribute to increased reward and reinforcement of vaping [11, 13], and present another target for intervention that incorporates the social needs of those who vape when developing regulatory policy and cessation approaches.

Our data also revealed the concept of “habitual vaping,” which refers to the automatic and often unconscious manner by which participants described their e-cigarette use. While current regulations on use of combustible nicotine products indoors prevent habitual smoking (i.e., smoking at any given time without thinking about it), such regulations are not always in place for vaping; others are able to vape indoors without notice (despite this not being allowed) due to the discrete nature of current vaping products. Moreover, the process of lighting a cigarette differs from that of using a vape (which requires no effort other than to bring the product to one’s mouth). Many participants expressed a clear recognition of their subconscious use of e-cigarettes, and reported that these use patterns likely stemmed from the convenience and ease in using e-cigarettes comparable to that of chewing gum or using a smartphone. Such availability and constant interaction with one’s vaping device was reported to produce feelings of immediate gratification and comfort (“adult pacifiers”) derived from the act of vaping. This constant use differs from traditional cigarettes which have a more definitive way of keeping track of the start and end of a smoking session (total number of cigarettes smoked), and the need to physically situate one’s self in a location where smoking is permitted. Participants also discussed their inability to track or quantify vaping intake, which again contrasts with combustible cigarette use, and which may make vaping a greater liability to dependent use patterns than cigarettes (particularly given the high levels of nicotine and ability to use these products anywhere). These findings highlight the risk in continued use of e-cigarettes over time, particularly of high nicotine-containing products, and the ability for e-cigarette users to develop dependence.

### Limitations

The following limitations should be considered when interpreting the current study findings. First, given the qualitative design of the study, we are limited in our ability to make strong statistical conclusions regarding temporality or causality. Second, our sample consisted of young adults who vape residing in Los Angeles county, and therefore results may not be generalizable to all young adults of a similar age group in different geographic locations of the U.S. Nevertheless, these data offer timely qualitative insights into experiences of e-cigarette dependency that have not been previously reported, and which have the potential to inform the development of stronger assessments and interventions of nicotine dependence specific to vaping.

## Conclusions

Young adults who vape reported indicators of e-cigarette dependence that are worthy of future research. While certain characterizations of e-cigarette dependence were shown to closely resemble that of nicotine dependence more broadly, the current study revealed unique insights of how young adults who vape experience e-cigarette dependence. Characteristics of e-cigarettes that differ from traditional cigarettes such as difficulty quantifying e-cigarette use, habitual vaping, social acceptability and norms, lack of vaping restrictions and accessibility, and immediate gratification and comfort felt from vaping devices may reinforce continued use and dependence liability.

## Supplementary Information

**Additional file 1: Supplemental Table 1.** Additional quotations by themes.

## Data Availability

All data generated or analyzed during this study are included in this published article [and its supplementary information files].
