# Supplementary material for: Characterizing symptoms of e-cigarette dependence: a qualitative study of young adults
Source: BMC Public Health. 2021 May 20;21:959. doi: 10.1186/s12889-021-10945-z (PMC8138971; doi:10.1186/s12889-021-10945-z)
Supplement: Supplementary file 1 — Additional file 1: Supplemental Table 1. Additional quotations by themes. [file 12889_2021_10945_MOESM1_ESM.docx]

| **Supplemental Table 1.** Additional quotations by themes | |
| --- | --- |
| **Themes** | **Quotations** |
| **Cravings and urgency to use** | I vape on the plane, but I just don’t vape as often and I try to go as long as I can… but like you know, it depends on the length of the flight. For a six hour… I went to New York recently it was like I can’t f*cking do this. Also when my vapes gonna die during the day like I didn’t charge it at night, I get… it’s overwhelming… anxiety. [ID34, white, male, age 23] |
| **Increased quantity and frequency of use to achieve desired effects** | And the past year I’ve been smoking [vaping] 6mg, so I’ve gone from a 3 to a 6, and now it’s kind of hard for me to put it down. [ID36, Hispanic/Latino, male, age 19] |
|  | It got to the point where it was not at the time that I would get the head rush, because it was always a rush system, that it took me either sleeping or not using for an extended period of time in order to get that head rush again. Like it’s not giving me any buzz or head rush, as people would say, because it’s already in my system. [ID28, white, male, age 19] |
|  | The tolerance for it goes up during the day um because I feel like that’s pretty normal even if you don’t smoke that often so you can take like 2 puffs in the morning right when you wake up and you’re like woah and then by the end of the day you’re watching TV and you’ve just been sucking on it for 5 minutes and you don’t feel it. [ID59, white, male, age 22] |
|  | It [nicotine consumption] definitely increased slowly because it’s easier to increase your tolerance and build on that and then just keep vaping more and more. I would say when I was starting I would take like 1 pod for a whole week and the next week it would be increased from that. [ID16, Asian, female, age 19] |
| **Unsuccessful quit attempts and withdrawal symptoms** | I’ve gone short periods of time, like flights, without vaping. And I got pretty uncomfortable and sick pretty quickly. [ID34, white, male, age 23] |
|  | I mean I told myself I’m not gonna do it anymore, and then it would be as I said, there’s never been a day where I haven’t hit one. But I have also like adopted this mentality that’s like I’ll only hit it if it’s going to give me that headrush cause if not there’s actually no point in hitting it. But it just never worked, it never happens. [ID28, white, male, age 19] |
|  | I know but I guess when or before you’re addicted and you start smoking you don’t realize because it’s easy to think like oh I’m mentally strong enough to just like stop, but you don’t realize that like even if you can do that it is hard to find a time to do that without it interfering with the things that really matter to you. [ID11, Asian, male, age 20] |
| **Greater nicotine consumption due to ease of accessibility and lack of vaping restrictions** | The need of it like even if I’m not stressed or anything I guess I will look for any little excuse just to go out and just smoke [vape] and um like sometimes I’m at work or something even if I’m doing something I’ll just go out of my way and just step out and just come back in really quick and I mean I can go places like I can go to a movie, go to a concert or something without even thinking about it but as soon as I get back to my car the very first thing I reach for before I even turn on the car. [ID14, Hispanic/Latino, male, age 21] |
|  | Honestly there was like a point where I would just be hitting it like every opportunity that presented itself, I’d be hitting it. Like every, obviously I was in school and stuff so I couldn’t just be hitting it, but every opportunity that I could I would be, so whether it be in the car, whether it be in my room, anywhere, and then when I would be doing my homework, I would just be sitting at my desk, hitting it as I was doing my homework. [ID28, white, male, age 19] |
|  | So when I wake up I get ready for work and then as soon as I’m driving to work I’ll take a hit and then I’ll get to work. As soon as I get out my car I have my vape in the locker and I take two quick hits um do a quick one or two hits and then I’m going to the freezer and then in about an hour and a half I’ll go on a break and I’ll be vaping on break. [ID10, Asian, male, age 22] |
| **Habitual vaping** | The Juuls you would hit a lot more constantly. Then you wouldn’t even hit it consciously, like without you knowing because it’s just so easy. But with a cigarette I feel like it’s kind of in your mind like when I’m going to smoke the cigarette and how much did I smoke today. So, I feel like in that way a Juul is a little more dangerous because people can’t really tell how much they’re taking. [ID35, Asian, male, age 20] |
| **Awareness of vaping dependency** | I think it’s just so familiar and also this nicotine can kind of have an effect… you’re addicted to it. I’m addicted to something that like, in theory, I want to stop doing but whenever I seriously contemplate quitting it’s scary. I feel kind of naked. [ID34, white, male, age 23] |
